# Alkohol als Kulturgut – eine historisch-anthropologische und therapeutische Perspektive auf Alkoholkonsum und seine soziale Rolle in westlichen Gesellschaften

**DOI:** 10.1007/s00103-021-03327-8

**Published:** 2021-05-14

**Authors:** Andreas Heinz, Laura S. Daedelow

**Affiliations:** Klinik für Psychiatrie und Psychotherapie, Charité – Universitätsmedizin Berlin, corporate member of Freie Universität Berlin and Humboldt-Universität zu Berlin & Berliner Institut für Gesundheitsforschung in der Charité – Universitätsmedizin Berlin, Charitéplatz 1, 10117 Berlin, Deutschland

**Keywords:** Sucht, Alkoholabhängigkeit, Droge, Krankheitskonzept, Prohibition, Addiction, Alcohol dependence, Drug, Disease concept, Prohibition

## Abstract

Alkohol ist die dominante Droge in westlichen Gesellschaften mit einer Geschichte, die sich vom Mittelalter über die Kolonialzeit bis in die Gegenwart zieht. Die historische Variabilität seines Konsums hat schon immer das Verständnis alkoholbezogener Probleme beeinflusst. Bis heute sind der öffentliche Diskurs über Suchterkrankungen und die Gestaltung des Versorgungssystems von Versatzstücken überholter Theorien geprägt, was zur Stigmatisierung und Diskriminierung betroffener Personen beitragen kann. Neben einem Überblick über die historische Entwicklung des Alkoholkonsums wird die soziokulturelle Diversität im Umgang und in der Einschätzung des Alkoholgebrauchs in westlichen Gesellschaften beleuchtet und ihre Relevanz für klinische Interventionen bewertet. Die Gründung einer bundesweiten Taskforce zur Gestaltung des Versorgungssystems wird empfohlen, um Kurzinterventionen und weitere wirksame Verfahren in der klinischen Praxis zu implementieren.

## Abstract (en)

Alcohol is the dominant drug in Western societies with a history that spans from the Middle Ages through the colonial times to the present day. The historical variability of its consumption has always influenced the understanding of alcohol-related problems. To this day, public discourse about addiction and the structure of the care system have been shaped by outdated theories, which can contribute to the stigmatization and discrimination of the affected persons. In addition to an overview of the historical development of alcohol consumption, the sociocultural diversity in dealing with and assessing alcohol use in Western societies is examined and its relevance for clinical interventions is assessed. A national task force to reform the healthcare system is recommended in order to fully implement short interventions and other effective procedures in clinical practice.

## Einführung

Alkohol ist die dominante Droge in westlichen Gesellschaften mit einer Geschichte, die sich weit vor die Zeit schriftlicher Aufzeichnungen erstreckt [1]. Im Folgenden soll die historische Variabilität seines Konsums und des damit zusammenhängenden Verständnisses alkoholbezogener Probleme erörtert werden, da sich Versatzstücke dieser Theorien bis heute im öffentlichen Diskurs finden und zur Gestaltung des Versorgungssystems, aber auch zur Stigmatisierung und Diskriminierung betroffener Personen beitragen können. Auf die historische Übersicht folgt eine Beschreibung der derzeitigen soziokulturellen Diversität im Umgang und in der Einschätzung des Alkoholgebrauchs in westlichen Gesellschaften, die bei allen Interventionen zu berücksichtigen ist. Ein Ausblick mit der politischen Forderung nach einer bundesweiten Expertengruppe zur Gestaltung des Versorgungsystems schließt den Überblick ab.

## Alkohol als Grundnahrungsmittel und die „Ernüchterung“

Im Mittelalter war Alkohol in Europa ein Nahrungsmittel, von dem pro Kopf (vom Kind bis zum Greis) etwa 3 Liter täglich konsumiert wurden [2]. Zum Verzehr gehörten damals aber auch Biersuppen, wobei ein Teil des Alkohols wahrscheinlich verkochte. Dennoch kann man sich einen Großteil der damals lebenden Menschen als permanent mehr oder weniger angetrunken vorstellen.

Eine „große Ernüchterung“ europäischer Gesellschaften kam zu Beginn des 17. Jahrhunderts mit dem transatlantischen Sklavenhandel und der Plantagenwirtschaft in der Neuen Welt auf, in dessen Folge sich neue Genussmittel wie Kakao, Kaffee und Zuckerrohr in der westlichen Welt verbreiteten. Unter unmenschlichsten Bedingungen wurden damals Sklaven aus Afrika nach Amerika verschifft, eingetauscht gegen Waffen, die Europa den Sklavenhändlern an der West- und Südwestküste Afrikas lieferte. Es wird geschätzt, dass ca. 10 Mio. Afrikaner in die Neue Welt verschleppt wurden, wo sie unter anderem die neuen Genussmittel anbauten, die dann unter ungeheuren Profitmargen nach Europa gelangten und hier zur „Ernüchterung“ der westlichen Gesellschaften beitrugen [2, 3]. In Europa befriedigten die neuen Genussmittel die Bedürfnisse des Adels und eines aufkommenden Bürgertums [4–7], welches der Nüchternheit und innerweltlichen Askese im Rahmen neuzeitlicher Rationalisierungen der Herrschafts- und Geschäftsverhältnisse eine wesentliche Bedeutung zuschrieb. Die westliche Aufklärung ist damit nicht unabhängig von der menschenverachtenden Sklaverei zu verstehen. Letztere findet aber wenig Beachtung im Räsonnement der Aufklärer.

Während der Aufklärung wird die „Trunksucht“ aber nicht einfach verpönt. Auch wenn es bereits vor dem 18. Jahrhundert Mäßigkeits- und Kontrollbestrebungen zur „Zivilisierung“ des Verhaltens beispielsweise in Klöstern gab [4], rechnete Kant die „Trunksucht“ nicht unter die pathologischen „Leidenschaften“, sondern zählte sie ebenso wie Spiel und Jagd (nur) zu den „Neigungen“. Leidenschaften waren für Kant immer dadurch gekennzeichnet, dass ein Mensch einen anderen Menschen „zum Mittel seiner Zwecke macht“, wie es bei Herrschsucht, Ehrsucht oder Habsucht der Fall ist, während Trunksucht nur als „Neigung“ zu bezeichnen sei, da daran kein anderer Mensch beteiligt ist [8]. Kants Konzeption ist bis heute von großem Interesse für die Definition der sogenannten Verhaltenssüchte, also der Frage, wann repetitive und für die betroffene Person letztlich schädlich wiederholte Verhaltensweisen als Suchterkrankung bezeichnet werden könnten. Denn hier kann mit Kant argumentiert werden, dass solche Verhaltenssüchte das mitmenschliche Gegenüber auf ein Mittel zum Zweck des Spielens reduzieren können [9]. Gegen Ende des 18. Jahrhunderts wurde die beginnende Industrialisierung von Disziplinierungen begleitet und befördert [10], die zur Pathologisierung von Konsumformen und -folgen beitrugen, die unter den neuen sozioökonomischen Bedingungen als gesellschaftlich disruptiv und persönlich schädlich angesehen wurden [4].

Etwa zur selben Zeit, im Jahr 1784, beschrieb der US-amerikanische Arzt und Humanist Benjamin Rush das starke, unwiderstehliche und unkontrollierbare Verlangen nach dem Alkoholkonsum [11]. Im Jahr 1813 wurde das *Delirium tremens* als Komplikation eines Alkoholentzugs von den britischen Ärzten Samuel Pearson und Thomas Sutton unabhängig voneinander erstmalig beschrieben [11]. Im 19. Jahrhundert gehörten sowohl das starke Verlangen nach dem Alkohol als auch die Kenntnis der Entzugssymptome zur Konzeption alkoholbezogener Probleme und wurden als die schädlichen Effekte des Alkoholkonsums genannt [12].

## Bewegungen zur Mäßigung des Alkoholkonsums und die Entstehung der Degenerationstheorie

In den Vereinigten Staaten von Amerika und in verschiedenen europäischen Ländern erstarkten im 19. Jahrhundert Bewegungen zur Mäßigung des Alkoholkonsums, u. a. weil Schnaps mit seinen ausgeprägten Effekten starke Verbreitung gefunden hatte. Historisch entstanden Bewegungen zur Mäßigung häufig nach einem Wechsel von traditionell etablierten Konsummustern zum Gebrauch höher konzentrierter Drogenzubereitungen, die gehäuft Abhängigkeitserkrankungen mit sich bringen können [13]. Anders als in vielen modernen Suchttheorien, die auf die Vulnerabilität des Individuums abheben, verstanden diese Bewegungen die Droge selbst als das entscheidende Problem: Demnach konnte jede Person, die zu viel trinkt, auch abhängig werden [14].

Demgegenüber entwickelte sich in der 2. Hälfte des 19. Jahrhunderts mit der „Degenerationstheorie“ eine zunehmende Fokussierung auf das betroffene Individuum. Die Degenerationstheorie, ursprünglich von dem französischen Psychiater Bénédict Augustin Morel [15] entwickelt, ging davon aus, dass leichte moralische Verfehlungen in der ersten Generation in der zweiten und den nachfolgenden zu immer schwereren, qualitativ andersartigen Erkrankungen führen können. Moralischer Verfall und Ausschweifungen in der ersten Generation könnten somit zu Trunksucht oder schwereren psychischen Erkrankungen in nachfolgenden Generationen führen, bis es schließlich zu ausgeprägten, auch körperlich nachweisbaren „Degenerationszeichen“ und Missbildungen komme. Dieses Konzept ging offenbar auf die damalige weite Verbreitung der Syphilis zurück, bei der eine (vermeintliche) moralische Verfehlung zur Infektion der betroffenen Person und, bei Infektion im Mutterleib, zu „Degenerationszeichen“ bei den so betroffenen Kindern führen kann. Die Degenerationstheorie nahm dabei an, dass Krankheiten über verschiedene Generationen hinweg einem erheblichen Gestaltwandel unterliegen [16].

Zu Beginn des 20. Jahrhunderts ließ sich das Konzept der Degeneration angesichts zunehmender empirisch fundierter Untersuchungen zur Erblichkeit einzelner Krankheitsbilder nicht länger aufrechterhalten, da diese ein getrenntes Risiko für unterschiedliche Erkrankungen und eben keine gemeinsame genetische Disposition für eine allgemeine „Degeneration“ nachweisen konnten [16]. Das Konzept der Degeneration bestimmte dennoch bis in die Zeit des 1. Weltkriegs hinein die Haltung weiter Teile der bürgerlichen Bevölkerung, da es die vermeintlich desaströsen Folgen auch kleiner Unachtsamkeiten und moralischer Verfehlungen mit imaginiert erblichem Schrecken verband. Degenerationsideen finden sich in Thomas Manns Roman *Buddenbrooks – Verfall einer Familie* (1901), aber auch bei Emil Kraepelin und anderen deutschen Psychiatern, die die Niederlage nach dem Ersten Weltkrieg und die demokratischen Aufstände antisemitisch interpretierten und als Degenerationszeichen deuteten [17, 18].

Ideologisch bot die Degenerationstheorie die Möglichkeit, Verarmung und soziale Missstände in der Industriegesellschaft nicht auf unhaltbare Lebensbedingungen, Unterernährung, soziale Perspektivlosigkeit und Verzweiflung zurückzuführen, sondern als vermeintlich unabänderliches individuelles Schicksal der Betroffenen zu definieren, das angeblich durch die unheilbaren degenerativen Anlagen bedingt sei.

## Die Alkoholprohibition in den Vereinigten Staaten von Amerika

Zu Beginn des 20. Jahrhunderts verlor die Degenerationstheorie aufgrund einer systematischer werdenden Erblichkeitsforschung an wissenschaftlichem Einfluss, während die Sorgen um die generell schädlichen Folgen des exzessiven Alkoholkonsums fortbestanden. Damit fokussierte sich die Diskussion um die schädlichen Folgen des Alkoholgebrauchs wieder verstärkt auf die Droge selbst. Die Mäßigungsbewegungen schafften es in den Jahren von 1919 bis 1933 in den Vereinigten Staaten von Amerika, eine generelle Prohibition, also das Verbot von Alkohol, durchzusetzen. Anders als es in vielen Hollywood-Filmen über diese Zeit angedeutet wurde, war diese Prohibition zuerst durchaus erfolgreich. Die Alkoholkonsummengen sanken deutlich. Allerdings nahm der illegale Alkoholkonsum mit der Zeit zu und kriminelle Organisationen wie die Mafia verdienten erheblich an der Beschaffung und Verbreitung des illegalisierten Getränks [19].

Die Prohibition scheiterte allerdings nicht an diesen illegalen Machenschaften, sondern an der anhaltenden Wirtschaftskrise in den 1930ern nach dem Zusammenbruch des Aktienmarktes. Damals wurde diskutiert, ob Einkommenssteuern einzuführen seien oder doch lieber der Alkohol wieder legal produziert werden könnte, da er damit besteuerbar wäre. Letztere Idee setzte sich nicht zuletzt unter der Einwirkung einflussreicher Ratgeber des damaligen Präsidenten durch [12].

## Zwangssterilisation und Ermordung alkoholkranker Menschen während der Diktatur der Nationalsozialisten 

Während einzelne Psychiater wie Kraepelin eine generelle Alkoholabstinenz forderten, verengte sich die öffentliche Diskussion nach Ende des Ersten Weltkriegs mehr und mehr auf die sogenannte Rassenhygiene. Entscheidende Befürworter waren unter anderem die Mediziner Eugen Fischer, Erwin Baur und Fritz Lenz [20], wobei Fischer und Lenz ihre damalige wissenschaftliche Autorität Studien an Opfern des deutschen Kolonialismus in Südwestafrika zu verdanken hatten. Dort wurde während des Aufstands der Herero und Nama von 1904 bis 1908 von der deutschen Militärführung der erste Genozid in der Geschichte des 20. Jahrhunderts verübt [21]. Die Zwangssterilisation schwer alkoholabhängiger Personen war von einzelnen Ärzten bereits vor der nationalsozialistischen Diktatur befürwortet worden, mit dem Machtantritt der Nationalsozialisten wurde sie umgesetzt. Wie viele alkoholabhängige Patientinnen und Patienten zudem während der nationalsozialistischen Diktatur im Rahmen der sogenannten Euthanasie ermordet wurden, ist nicht bekannt [22].

## Die Anonymen Alkoholiker und der Beginn eines modernen Krankheitskonzeptes

In den Vereinigten Staaten von Amerika folgte auf das Scheitern der Alkoholprohibition eine Trendwende in der Konzeptualisierung des problematischen Alkoholkonsums, da sich ein allgemeingültiges Verbot gerade als politisch nicht durchsetzbar erwiesen hatte. Stattdessen verschob sich die Erklärung der Alkoholprobleme auf das betroffene Individuum, das – anders als zu Zeiten der Degenerationstheorien – jetzt eher als besonders verletzlich beziehungsweise empfindlich verstanden wurde. Zu dieser Verschiebung des Diskurses trug nicht zuletzt das Engagement der Selbstorganisationen der Betroffenen bei, so auch das der „Anonymen Alkoholiker“, die zum Ende der 1930er-Jahre gegründet worden waren. Damit gewann eine einerseits individualisierende, andererseits aber unterstützende und zumindest ansatzweise anerkennende Haltung gegenüber alkoholabhängigen Patientinnen und Patienten an Gewicht [12].

Demgegenüber wurden die körperlich sichtbaren Auswirkungen exzessiven Alkoholkonsums bspw. im Sinne der Entzugssymptomatik oder des schweren Delirium tremens in den 30er- und 40er-Jahren des letzten Jahrhunderts weitgehend ignoriert. Erst Mitte der 1950er-Jahre wurden Krampfanfälle und Delirium tremens von der Weltgesundheitsorganisation als wichtige Symptome des Alkoholentzugs thematisiert [23]. Toleranzentwicklung und Entzugssymptomatik bei plötzlicher Unterbrechung der Alkoholzufuhr wurden damit zu festen Bestandteilen eines modernen Krankheitskonzepts, so bei Jellinek [24] und Edwards [23]. In Deutschland wurde ein modernes Krankheitskonzept unter anderem von Feuerlein [25] propagiert, der zudem darauf hinwirkte, dass alkoholabhängige Patientinnen und Patienten dieselben Rechte zur medizinischen Behandlung haben sollten, wie alle anderen Kranken im deutschen Gesundheitssystem. Es war allerdings erst 1968, als der volle Versicherungsschutz für alkoholbezogene medizinische Behandlungen gesetzlich verbindlich verankert wurde [13].

## Sucht oder Abhängigkeit – Pendelbewegungen im modernen Krankheitskonzept

Seit Aufkommen moderner Krankheitsklassifikationen der US-amerikanischen Fachgesellschaft der Psychiaterinnen und Psychiater (American Psychiatric Association, APA; [26]) und der Weltgesundheitsorganisation (WHO; [27]) lassen sich Pendelbewegungen zwischen einer Fokussierung des Krankheitsverständnisses einerseits auf das starke Verlangen und die Kontrollminderung im Umgang mit der Droge (Sucht) und andererseits auf Toleranzentwicklung und Entzugssymptomatik (Abhängigkeit) beobachten. Was als theoretischer Streit anmuten kann, hat durchaus relevante sozialpolitische Implikationen.

Edwards [23] fokussierte auf die Alkoholentzugssymptomatik, die sich unter anderem nach einer Untersuchung bei seinen Studierenden bei jeder Person zeigte, die über längere Zeit hochprozentigen Alkohol konsumiert hatte. Edwards’ Fokus lag damit auf den Wirkungen des Alkohols auf das zentrale Nervensystem, wo er neurobiologische Anpassungsvorgänge fand, die unter den Bedingungen des fortgesetzten Alkoholkonsums zur Herstellung eines neuen Gleichgewichts, einer sogenannten Homöostase führen. Wird der Alkoholkonsum plötzlich unterbrochen, kommt es dementsprechend zu einem Ungleichgewicht in der zentralnervösen Erregungsverarbeitung und damit zu Entzugserscheinungen.

Der Schwerpunkt der Diskussion verschob sich somit zu einer „somatisch verankerten Drogenabhängigkeit“, die oft fälschlich auch als „körperlich“ bezeichnet wird. Falsch ist diese Bezeichnung dann, wenn der körperlichen Abhängigkeit eine vermeintlich unkörperliche, „psychische“ Abhängigkeit entgegengestellt wird. Denn Letztere bezieht sich auf Symptome wie das Alkoholverlangen und die Kontrollminderung, denen ebenso wie Entzugssymptomen neurobiologische Korrelate im zentralen Nervensystem zugeordnet werden können, beispielsweise im sogenannten dopaminergen Belohnungssystem und im präfrontalen Kortex [28].

Mit dem Begriff der Sucht (im Englischen meist mit der Bezeichnung „addiction“ thematisiert) fokussierte sich die Diskussion demgegenüber auf das starke Verlangen und die erlebte Kontrollminderung der Betroffenen. Bei dieser Sichtweise können allerdings die alten Degenerationshypothesen wieder anklingen, indem den erkrankten Personen unterstellt wird, dass es ihnen an „Willensstärke“ mangele bzw. dass sie „zu impulsiv“ seien und sich somit zu sehr von ihren vermeintlich „primitiven“ Gefühlen und ihrem Verlangen steuern ließen [9].

Während die International Classification of Diseases (ICD-10) von 2004 als Krankheitsklassifikation der WHO [27] noch unter dem Einfluss von Suchtforschern wie Edwards und anderen das Konzept der Abhängigkeit („dependence“) vertritt, widmet sich die APA mit dem Diagnostic and Statistical Manual (DSM‑5, 2013; [26]) dem Suchtkonzept und gibt den Begriff der „dependence“ ganz auf. Sie spricht stattdessen von „Substanzgebrauchsstörungen“, die sich bruchlos vom schädlichen Konsum bis zur schweren Gebrauchsstörung entwickeln sollen. Allerdings wurde diesem Konzept vorgeworfen, dass es den schlecht definierten Begriff des „schädlichen Gebrauchs“ mit dem klinisch relativ gut abgrenzbaren Begriff der „Abhängigkeit“ vermengt [29].

Der schädliche Gebrauch ist deswegen schwer zu klassifizieren, weil Fragen der Illegalisierung einer Droge zu sozialen Problemen und Einschränkungen führen können, die bei einer Gesetzesänderung nicht mehr gegeben wären. So sind der Zeitaufwand zur Beschaffung einer Droge und die negativen sozialen Folgen deren Konsums nicht unabhängig vom gesetzlichen Umgang und der Strafverfolgung beim Gebrauch einer Droge. Am DSM‑5 wurde dementsprechend kritisiert, dass ein zu breiter Begriff der Substanzgebrauchsstörung zur Pathologisierung größerer Bevölkerungsgruppen führen kann und dass stattdessen eine Fokussierung auf die schwerer betroffenen Individuen notwendig sei, da nach wie vor ein Großteil dieser Patientinnen und Patienten nicht adäquat versorgt wird [29, 30].

## Soziokulturelle Differenzen im Umgang mit der Droge Alkohol

Professionelle, die sich im medizinischen Hilfesystem engagieren, hoffen oft, dass Patientinnen und Patienten die von ihnen gebotenen Informationen und Ratschläge unmittelbar verstehen und umsetzen. Nicht erst die Auseinandersetzungen um die Coronapandemie zeigen, dass dies nicht immer der Fall ist. Seit Jahrzehnten weist dementsprechend eine sozialanthropologische Forschung (im deutschen Sprachraum meist unter den Begriffen „Ethnologie“ oder „kulturelle Diversität“ fungierend) darauf hin, dass generelle medizinische Informationen und Krankheitsmodelle von den Betroffenen auf Grundlage ihrer bisherigen Überzeugungen höchst unterschiedlich verarbeitet werden [31]. Diese individuelle Variabilität wird unter dem Namen der „Erklärungsmodelle“ thematisiert, die von jeder einzelnen Person unter dem Einfluss einer Vielzahl zeitgenössischer und überlieferter Theorien, kultureller Traditionen und Praktiken, sozialer Diversität, geschlechtlicher Zuordnung und öffentlich diskutierter Überzeugungen ausbildet werden. Selbst wenn Professionelle und Betroffene dieselben Wörter gebrauchen, müssen diese Ausdrücke deshalb im Kontext der individuell unterschiedlichen Erklärungsmodelle nicht dasselbe bedeuten.

Bezüglich kultureller Diversität beobachteten Penka und andere [32], dass Jugendliche mit türkischem Migrationshintergrund in Mannheim viele Begriffe der „körperlichen“ Abhängigkeit als ungeeignet bezeichneten, um eine Suchterkrankung zu charakterisieren. Andererseits entsprach das Verständnis der Gefährlichkeit von legalen und illegalen Drogen bei den Jugendlichen mit türkischem Migrationshintergrund viel eher dem medizinischen Schulwissen, als dies bei den in Mannheim ebenfalls untersuchten Jugendlichen mit traditionell deutschem Hintergrund der Fall war. Denn Letztere neigten ebenso wie Jugendliche aus Spätaussiedlerfamilien aus der ehemaligen Sowjetunion dazu, die Gefährlichkeit der Drogen Alkohol und Tabak gegenüber illegalen Drogen zu vernachlässigen [32]. Weiterführende Untersuchungen bei Menschen aus Berlin und Istanbul ergaben, dass soziale Unterschiede sich noch weit stärker auf das Verständnis psychischer Erkrankungen auswirken als unterschiedliche kulturelle Traditionen: Bildungswege und sozialer Status hatten besonders ausgeprägten Einfluss auf die Ausgestaltung des individuellen Krankheitsverständnisses [33].

Die einfache Übersetzung im deutschen Sprachraum etablierter Aufklärungsbroschüren würde demnach nicht genügen, um Menschen mit einem sozial oder kulturell different geprägten Vorverständnis zu erreichen. Auch in der jeweiligen therapeutischen Kommunikation ist es hilfreich, bei allen Patientinnen und Patienten differenziert nachzufragen, wie die professionell bereitgestellte Information verstanden und interpretiert wird.

## Heutiges Krankheitsverständnis und Ausblick

Die Auseinandersetzung um kulturell tief verankerte und allgegenwärtige Drogen wie den Alkohol und insbesondere um die Konzeptualisierung des Krankheitsverständnisses prägt bis heute den gesellschaftlichen und den institutionellen Umgang. Soziale Ausgrenzung und Stigmatisierung von Suchtkranken findet bis heute statt, sichtbar auch daran, dass von den Krankenkassen bis heute regelhaft nur die Alkoholentgiftung, nicht aber die weitergehende Behandlung der Suchterkrankung finanziert wird. Letztere wird als Rehabilitation dem Verantwortungsbereich der Rentenversicherung zugeschrieben. Dadurch kommt es beim Übergang von der akuten Entgiftung in die (rehabilitative) Entwöhnung noch immer häufig zu Behandlungsabbrüchen.

Nach wie vor findet sich zudem ein Großteil alkoholabhängiger Patientinnen und Patienten nicht in spezialisierter Behandlung, sondern nur im Kontakt mit dem auf somatische Erkrankungen ausgerichteten Gesundheitssystem [34]. Trotz Vorliegen wirksamer Kurzinterventionen, die eine Vielzahl abhängiger Patientinnen und Patienten erreichen könnten, werden diese immer noch allzu selten angewendet [34]. Gerade bei kulturell so tief verankerten Drogen wie dem Alkohol gibt es also offenbar eine Tendenz der Gesellschaft, den Konsum zu verharmlosen und die Schuld für alkoholbezogene Probleme inklusive der Entstehung einer Abhängigkeitserkrankung auf die Betroffenen zu projizieren. Dies zu kritisieren heißt nicht, dass eine genetisch verankerte Empfindlichkeit für die Entstehung einer Alkoholabhängigkeit ignoriert werden muss.

Nach Untersuchungen von Schuckit und anderen [35] besteht diese genetische Disposition allerdings in etwas so relativ „Harmlosem“ wie der Fähigkeit, größere Mengen Alkohols ohne akut auftretende unangenehme Auswirkungen zu konsumieren. Wer also andere „unter den Tisch“ trinken kann, fühlt sich vermeintlich gegen die Gefahren des Alkoholkonsums gefeit und neigt deshalb leider dazu, auch langfristig vermehrt Alkohol zu konsumieren, woraus sich ein deutlich erhöhtes Risiko für die Entstehung einer Alkoholabhängigkeit ergibt. Dieser genetischen Vulnerabilität lassen sich auch neurobiologische Korrelate bspw. im Bereich der monoaminergen Neurotransmission zuordnen [28]. Eine zumindest teilweise genetisch verankerte Toleranz gegenüber den akuten Alkoholwirkungen ist aber nur dann bedenklich, wenn Alkoholkonsum in einer Gesellschaft weitverbreitet und „Trinkfestigkeit“ sozial anerkannt ist. Genetische Faktoren wirken sich wahrscheinlich nie unabhängig von den Umweltbedingungen aus, in die eine Person hineingeboren wird.

Will man die ausgeprägte Problematik des Alkoholkonsums in westlichen Industriegesellschaften angehen, müssen Vorurteile und die Stigmatisierung von Suchtkranken ebenso thematisiert werden wie institutionelle Mechanismen, mit denen Suchtkranke sozial ausgeschlossen oder benachteiligt werden. Hierzu sollte eine politisch unterstützte Expertengruppe (Taskforce) mit Beteiligung von Verbänden der Betroffenen, Angehörigen und Professionellen etabliert werden. Ihre Aufgabe sollte es sein, ähnlich wie einst die Psychiatrie-Enquête, das gesamte Behandlungsangebot zu sichten und zu bewerten. Damit könnte auch die längst fällige, breite Implementierung von Kurzinterventionen und weiteren wirksamen Verfahren in Allgemeinpraxen, auf chirurgischen Unfallstationen, im psychiatrischen Konsiliardienst und in der Heimversorgung durchgesetzt werden.
